# Strengthening mental health among university students

**DOI:** 10.3389/fpsyg.2025.1689173

**Published:** 2025-11-06

**Authors:** Naiara Ozamiz-Etxebarria, Nahia Idoiaga Mondragon, Natalia Tsybuliak

**Affiliations:** 1 Department of Developmental and Educational Psychology, University of the Basque Country, Bilbao, Spain; 2 Department of Applied Psychology and Speech Therapy, Berdyansk State Pedagogical University, Zaporizhzhia, Ukraine

**Keywords:** student mental health, higher education, proactive mental health strategies, inclusive education, institutional culture, psychological wellbeing, crisis resilience

## Introduction

The COVID-19 pandemic amplified long-standing vulnerabilities in university students' mental health, exposing critical weaknesses in institutional support systems (Son et al., 2020; Segú-Odriozola, 2025). Even before the crisis, students faced heavy academic workloads, transitional life stages, and financial pressures, factors that, in unsupportive environments, heightened the risk of psychological strain (Grimmond et al., 2020; Zahedi et al., 2022). The pandemic acted as a catalyst, magnifying these pressures through the abrupt loss of in-person peer networks, reduced access to campus-based services, and reliance on remote learning environments often lacking adequate psychosocial support (Elmer et al., 2020; Khoshaim et al., 2020; Sundarasen et al., 2020). This was not merely a temporary disruption but a sustained weakening of the social and institutional structures essential for academic engagement and personal wellbeing (Hamza et al., 2021).

While this perspective takes a global view, it also recognizes that political contexts, resources, and institutional structures vary widely. Such differences influence both the design and effectiveness of mental health programming in higher education. By integrating case studies from diverse settings, this paper aims to highlight approaches with potential for adaptation across contexts, rather than prescribing a one-size-fits-all solution.

Against this backdrop, the mental health impacts of the pandemic have endured worldwide. A systematic review and meta-analysis by Li et al. (2021) reported that approximately 39% of university students experienced symptoms of depression and 36% reported symptoms of anxiety during the pandemic, substantially higher than pre-pandemic estimates around 20–25%. Longitudinal data from the United Kingdom indicate that these elevated rates persisted for at least 12 months beyond the acute phase (Allen et al., 2023). While prevalence varied across regions, disciplines, and demographic groups, the overarching message is clear: higher education is facing a persistent mental health crisis.

Despite growing recognition of the problem, many institutional responses remain fragmented, reactive, and insufficiently embedded in the fabric of university life. In some universities, support is limited to short-term counseling or crisis hotlines, used only after students show serious distress (Grubic et al., 2020; Son et al., 2020). Others have introduced short wellness campaigns or peer-support activities, but without ongoing funding these often lose impact over time (Lister et al., 2022; Lea et al., 2023). At the other end of the scale, a small but growing group of universities have adopted “whole-university” models that include mental health in teaching, staff training, campus design, and leadership policies (Priestley et al., 2022). Even so, these broader approaches often lack coordination between departments, meaning that support can be uneven and dependent on individual staff rather than clear university policy. Across this spectrum, the absence of systemic, preventive strategies leaves higher education vulnerable not only to ongoing mental health deterioration but also to future large-scale disruptions, whether from another pandemic, global economic instability, or armed conflicts such as those in Ukraine or Gaza.

This opinion draws on pandemic-era lessons to assess current approaches and propose a systemic, participatory agenda for action. Framing mental health as a shared responsibility, it advances a three-pronged strategy for higher education:

- Shifting from reactive crisis management to proactive, culture-wide approaches.
- Embedding psychological wellbeing as a foundation for learning, equity, and personal development.
- Promoting inclusive, co-created solutions involving students, faculty, and staff.

In doing so, it aims to contribute to the global conversation on building healthier, more inclusive, and crisis-resilient learning environments. While universities have responsibilities across many dimensions of health, we foreground mental health because it is closely tied to the core academic mission. Mental health and wellbeing underpin attention, memory, motivation, and a sense of belonging, which are capacities essential for learning and persistence. During and after the pandemic, elevated depression and anxiety were associated with sustained academic strain, reinforcing this linkage (Li et al., 2021; Allen et al., 2023). Integrating mental health into teaching, assessment, student services, and campus life therefore advances educational quality and equity rather than competing with them. This emphasis does not replace physical health or general wellbeing; instead, it recognizes mental health as a key pathway through which universities fulfill their teaching and inclusion mandates.

## Lessons from the pandemic

The pandemic intensified vulnerabilities in student mental health that had long been present but insufficiently addressed. In Spain, for example, large-scale surveys have documented marked increases in emotional exhaustion, anxiety, and depressive symptoms among university students (Soler et al., 2025; González Rico et al., 2024; Ozamiz-Etxebarria et al., 2020). Similar patterns have emerged worldwide (Li et al., 2021), confirming that this is a systemic challenge rather than a local anomaly.

Universities were often unprepared for the surge in psychological distress. Responses tended to be piecemeal and reactive, constrained by long wait times, insufficient staffing, and limited service capacity (Grubic et al., 2020; Son et al., 2020; Hossain et al., 2020). Persistent stigma further discouraged help-seeking, particularly among marginalized groups (Salerno et al., 2020). The pandemic revealed the absence of integrated, preventive mental health frameworks, underscoring the need for proactive, long-term strategies embedded in institutional culture (Zhai and Du, 2020).

Students have been clear about the reforms they view as essential: integrating mental health topics into curricula, increasing the visibility and accessibility of services, and equipping academic staff with psychological first aid skills. Research linked to the University Mental Health Charter advocates a “whole university approach” that addresses both cultural and structural dimensions (Priestley et al., 2022). Training in mental health literacy, through initiatives such as Mental Health First Aid, has been shown to improve knowledge, attitudes, and confidence in responding to distress (Llopis et al., 2023). Supportive faculty–student relationships also act as buffers against stress (Payne, 2022).

One consistently identified protective factor is social support. Perceived support from peers, family, or university staff correlates strongly with lower stress, anxiety, and depression (Howlader et al., 2024; Cao et al., 2024; Liu et al., 2021). This aligns with Lazarus and Folkman's (1984) transactional theory of stress and coping, which emphasizes the role of coping resources in shaping outcomes. Universities that actively foster these connections, through peer-to-peer initiatives, mentoring schemes, and community engagement, can strengthen resilience and normalize help-seeking.

Some promising models integrate these insights. For example, the Erasmus+-funded Aware Project (2025) trains staff, develops educational resources, and creates participatory tools for prevention and early identification. Involving students in the design of interventions allows us to go beyond top-down approaches, fostering trust and relevance (Lister et al., 2022). Strategies such as participatory workshops have been shown to improve the adaptation and acceptability of mental health tools (Lira et al., 2023), while student-led peer platforms have helped reduce stigma and increase accessibility (Lea et al., 2023). Universities varied widely in their capacity to sustain such efforts: some developed coordinated, campus-wide strategies, while others relied on isolated actions or short-term measures, leading to notable disparities in support quality.

Complementing clinical services, recreational activities, sports, arts, student societies, and volunteering can strengthen peer networks and perceived social support—well-established protective factors against stress, anxiety, and depression (Elmer et al., 2020; Liu et al., 2021; Cao et al., 2024; Howlader et al., 2024). A whole-university approach should therefore connect curricular provision and counseling with these co-curricular spaces, making them intentional, inclusive, and well-signposted.

To illustrate how evidence-based, well-coordinated initiatives can be implemented under diverse political, cultural, and resource conditions, we highlight several programmes from different national contexts. The University of California, Los Angeles introduced the STAND stepped-care programme, screening thousands of students and providing tiered digital and clinical interventions that significantly reduced depression and anxiety symptoms and lowered suicide-risk severity among those receiving higher-intensity care (Wolitzky-Taylor et al., 2023). In Italy, the University of Foggia's online one-to-one counseling service was linked to marked increases in wellbeing and decreases in stress, anxiety, and distress (Celia et al., 2022). The University of Coimbra in Portugal implemented a stepped-care model with structured triage and monitoring, improving service flow and access during peak demand (Marques et al., 2024). In the United Kingdom, Newcastle University's CBT-based student clinic maintained recovery rates comparable to pre-pandemic levels (~47% by NHS criteria) while delivering therapy remotely, and students reported high satisfaction with accessibility and continuity of care (Robinson et al., 2024; Newcombe et al., 2025). Together, these cases show that integrated, well-evaluated strategies can sustain and even enhance mental health support in crisis contexts, offering scalable models for other institutions.

Nevertheless, progress remains uneven. Three structural shortcomings continue to limit the effectiveness and reach of current initiatives:

Gap 1—Over-reliance on late-stage clinical interventions. Many systems still treat student mental health narrowly in clinical terms, focusing on therapeutic intervention only after symptoms become severe. During the pandemic, lack of early intervention infrastructure meant universities often defaulted to fragmented crisis management (Son et al., 2020). While counseling and psychiatric services remain essential, they are often under-resourced and disconnected from the academic environment, reinforcing the perception that mental health is peripheral to the university's mission (Grubic et al., 2020; Zhai and Du, 2020).

Gap 2—Limited inclusivity in provision. Institutional strategies often fail to account for the diversity of the student population. Students with disabilities, LGBTQIA+ individuals, first-generation learners, and those from migrant or low-income backgrounds face distinct and intersecting stressors. Generic campaigns rarely meet these needs. As Salerno et al. (2020) emphasize, sexual and gender minority students encounter “compounded risks for poor mental health” due to intersecting stigma and pandemic-related challenges. Equity, cultural competence, and tailored provision must be central to policy (Segú-Odriozola, 2025; Hamza et al., 2021).

Gap 3—Staff preparedness and support. Academic staff are often first-line contacts for students in distress but frequently lack the training, confidence, or time to respond effectively (Payne, 2022; Lopatina et al., 2024). Capacity-building initiatives such as Mental Health First Aid can significantly improve staff readiness (Llopis et al., 2023), but uptake is inconsistent without institutional mandates and protected time.

The key lesson is clear: universities are not merely sites of academic instruction but complex social ecosystems whose health directly shapes student wellbeing (Elmer et al., 2020). Embedding mental health into institutional culture is essential to address current challenges and prepare for future crises.

## Discussion: from lessons to action

In practice, connecting mental health to the university mission means aligning pedagogy, student services, and co-curricular life so academic policies, staff development, and campus communities work together to reduce avoidable strain and bolster social support (Priestley et al., 2022; Lister et al., 2022; Lea et al., 2023). Concretely, embed a small set of classroom practices that lower cognitive load and strengthen belonging, transparent assessment maps and staggered deadlines, early low-stakes tasks that scaffold toward high-stakes work, structured peer activities with clear roles, and brief 3–5 min check-ins that signpost a single digital “front door” for support, without medicalising the classroom (Priestley et al., 2022; Lister et al., 2022). A short syllabus statement that normalizes help-seeking and sets flexibility windows aligns classroom norms with campus services, while basic gatekeeper training (Recognize–Support–Refer) equips staff to respond and refer, not treat (Payne, 2022; Llopis et al., 2023). These moves connect curricular learning with co-curricular networks (societies, sport, arts, and volunteering) that buffer stress through social ties (Elmer et al., 2020; Lea et al., 2023). Given links between elevated anxiety/depression and academic strain during and after the pandemic, this pedagogy is mission-relevant (Li et al., 2021; Allen et al., 2023). Progress can be tracked with routine indicators—on-time and early-assessment completion, DFW/withdrawal rates, progression, and brief belonging items—keeping evaluation focused on learning outcomes.

We propose a three-pronged, evidence-informed strategy, supported by sustained institutional capacity building, to embed mental health into the core mission of higher education:

1. Training and awareness for academic and administrative staff. Universities should invest in structured, ongoing professional development so staff can recognize early signs of psychological distress (e.g., persistent withdrawal, mood changes, declining performance) and respond in an informed, timely, and non-stigmatizing manner. For example, making “Mental Health First Aid” part of mandatory induction for new staff, with annual refreshers, can improve recognition of distress signals and referral pathways. Embedding “trauma-informed” principles into training for academic and administrative personnel is essential for engaging with vulnerable student populations, including those affected by crisis, displacement, or discrimination (Payne, 2022; Llopis et al., 2023).
2. Participatory mental health interventions. Co-designed initiatives, such as peer mentoring programs, student-led support platforms, and mental health literacy campaigns, tend to increase relevance, trust, and uptake, while normalizing help-seeking and reducing stigma (Lister et al., 2022; Lea et al., 2023). For instance, “Student Wellbeing Ambassadors” at several UK universities run drop-in listening sessions, organize wellbeing events, and signpost peers to professional services. Moderated digital peer communities also extend support to online and hybrid learners. Strong peer networks are especially valuable in mitigating social isolation in both crisis and routine contexts (Elmer et al., 2020).
3. Inclusion and equity-oriented frameworks. Strategies must address the specific needs of marginalized and at-risk groups, students with disabilities, LGBTQIA+ students, first-generation learners, and those from migrant or low-income backgrounds (Salerno et al., 2020). This can include targeted mentorship for first-generation students, accessible counseling (e.g., interpreters, assistive tech), and culturally responsive resources for international and refugee students. An “intersectional” approach, pairing gender-sensitive policies with anti-racist and “anti-ableist” practices, helps remove overlapping barriers. As Salerno et al. (2020) note, sexual and gender minority students face compounded risks for poor mental health, which generic campaigns often fail to address.

This framework complements rather than replaces clinical services. Its goal is to cultivate institutional cultures where mental health is recognized as a shared responsibility and a prerequisite for both academic success and personal development. Universities that simply return to pre-pandemic norms risk reinstating systems that proved fragile under stress (Grubic et al., 2020). Instead, they should seize this moment to redefine what constitutes a healthy, resilient learning environment.

Such transformation directly supports the United Nations Sustainable Development Goal 4 (Quality Education) (United Nations, 2015) and aligns with UNESCO's (2023) call for inclusive, adaptive, and resilient educational systems that treat wellbeing as foundational to learning. Beyond policy alignment, this is a strategic imperative for higher education in an era of recurring crises. Institutions that act decisively can position themselves not only as places of academic instruction but also as environments where students gain the emotional resources, social connections, and sense of belonging essential for thriving amid uncertainty.

## Feasibility and division of responsibilities

Recognizing funding and staffing constraints, we distinguish between actions that fall squarely within the university's remit and those better delivered through cross-sector partnerships. The aim is to embed a minimum viable package on campus while leveraging external capacity for specialized care.

(1) Core university remit (integrated into existing roles and processes)

- Pedagogy and assessment: reduce avoidable academic strain (transparent workload planning, staggered deadlines, flexible assessment pathways); include brief syllabus statements that normalize help-seeking.
- Early identification and signposting: offer light-touch screening or check-ins at key transition points; create a single, well-advertised digital “front door” that routes students to self-help, peer support, or clinical referral as appropriate.
- Staff capability: deliver brief, recurring gatekeeper/mental-health-first-aid training via a train-the-trainer model; provide trauma-informed interaction basics for academic and administrative staff.
- Social belonging: intentionally link counseling/advising with co-curricular spaces (sports, arts, societies, and volunteering) to strengthen peer networks—an established protective factor.
- Measurement for improvement: track 3–5 feasible indicators (e.g., wait times, utilization, brief outcome measures, training coverage, sense of belonging) and review each term.

(2) Shared responsibilities via formal partnerships (MoUs/SLAs)

- Stepped-care interfaces: set clear referral pathways to community and tele-mental-health providers, with shared protocols for risk escalation and after-hours cover.
- Specialist provision: engage culturally specific NGOs (for groups facing compounded risks), language/interpretation services, and partner-delivered group psychoeducation on campus or online.
- Workforce extension: use supervised placements for psychology/social work/psychiatric-nursing trainees to expand capacity at low cost, with partners providing clinical governance.

(3) External system responsibilities (outside university scope)

24/7 crisis lines and mobile crisis response, specialist psychiatry, inpatient/outpatient care, and long-term psychotherapy. Universities coordinate and signpost—do not duplicate.

Minimum viable package (resource-light starting point)

- A single navigation hub for mental health and wellbeing.
- Two hours per semester of basic gatekeeper training for all student-facing staff.
- Short transition-point check-ins (e.g., first year, return from leave, and placement years) with scripted signposting.
- Coordination with existing clubs/societies to run low-cost, inclusive belonging activities.
- A standing MoU with at least one external provider for rapid escalation and after-hours coverage.

Financing and incentives

- Align mental-health metrics with existing institutional goals (retention, progression, and equity) to justify reallocation.
- Use student placements, philanthropy, and public/insurance reimbursement where applicable (e.g., brief therapies via partners).
- Prefer consortia procurement and open-source/self-help resources before new spend.

Evidence from stepped-care and partnership models indicates improved access and outcomes without building parallel clinical infrastructures (Wolitzky-Taylor et al., 2023; Marques et al., 2024; Robinson et al., 2024). Framing actions through this division of labor and phasing them keeps the approach feasible for under-resourced institutions while maintaining fidelity to the university's core mission.

## Conclusion

The COVID-19 pandemic revealed that student mental health is a structural issue for higher education, not a temporary disruption. It demonstrated that resilience is not built in crisis but through sustained cultural and institutional commitments long before disruption occurs. Universities that fail to embed mental health into their core mission will remain vulnerable to academic, economic, and social fallout in future emergencies, whether triggered by global health crises, economic instability, or armed conflict.

The way forward is to shift from seeing mental health as an adjunct service to treating it as a precondition for educational quality and equity. This requires long-term investment, governance-level commitment, and the inclusion of student voices in shaping support systems. The three pillars outlined in this opinion, moving from reactive crisis management to proactive, culture-wide approaches; embedding wellbeing as a foundation for learning and equity; and promoting inclusive, co-created solutions, provide a framework for action that is adaptable across contexts.

This perspective is not a fixed prescription but an invitation for constructive dialogue among students, faculty, administrators, and policymakers on how to bring these pillars to life in practice. Such dialogue is essential to co-create universities that are not only centers of knowledge but also communities of belonging and resilience, places where learning is inseparable from emotional support and where students gain the resources, relationships, and sense of belonging necessary for growth. In a century likely to be defined by recurring crises, the choice is clear: return to pre-pandemic fragility or build higher education systems capable of sustaining both academic excellence and human flourishing, in stability and in uncertainty alike.
